# Supplementary material for: Hybrid Models and Biological Model Reduction with PyDSTool
Source: PLoS Comput Biol. 2012 Aug 9;8(8):e1002628. doi: 10.1371/journal.pcbi.1002628 (PMC3415397; doi:10.1371/journal.pcbi.1002628)
Supplement: Text S4 — Complete source code for the PyDSTool package (version 0.88.120504). Includes API documentation and help files linking to web pages. This file is identical to the current public release on Sourceforge.net. (ZIP) [file pcbi.1002628.s004.zip › PyDSTool/html/PyDSTool.Toolbox.optimizers.criterion-module.html]

xml version="1.0" encoding="ascii"?


PyDSTool.Toolbox.optimizers.criterion


| Home | Trees | Indices | Help | PyDSTool |
| --- | --- | --- | --- | --- |

Package PyDSTool ::
Package Toolbox ::
Package optimizers ::
Package criterion

[hide private]

[frames] | no frames]

# Package criterion

source code

Module containing every criteria for converge test

Functions :

- crietrion() creates a composite criterion

Criteria :

- IterationCriterion
  - stops when the iteration limit is reached
- MonotonyCriterion
  - stops when the cost rises again
- RelativeValueCriterion
  - stops when the relative value error is below a certain level
- AbsoluteValueCriterion
  - stops when the absolute value error is below a certain level
- RelativeParametersCriterion
  - stops when the relative parameters error is below a certain level
- AbsoluteParametersCriterion
  - stops when the absolute parameters error is below a certain level
- GradientCriterion
  - stops when the gradient is below a certain level

Composite criteria :

- OrComposition
  - returns True if one of the criteria returns True
- AndComposition
  - returns True if all the criteria return True

Information criteria :

- AICCriterion
  - stops when the cost function increases again
  - is dependent of the number of parameters if it changes
- ModifiedAICCriterion
  - Identical to AICCriterion except that a number of increases are
    tolerated


|  |  |
| --- | --- |
| Submodules | [hide private] |

- **PyDSTool.Toolbox.optimizers.criterion.composite\_criteria**: *Composite criteria allow to use several criteria together, with
  and/or composition*
- **PyDSTool.Toolbox.optimizers.criterion.criteria**: *A list of standard convergence criteria based on the number of
  iterations, the last values taken by the cost function and the
  associated points*
- **PyDSTool.Toolbox.optimizers.criterion.facilities**: *Proposes a way to create a composite criterion*
- **PyDSTool.Toolbox.optimizers.criterion.information\_criteria**

  


|  |  |
| --- | --- |
| Classes | [hide private] |

RelativeValueCriterion  
The relative criterion stops the optimization when the relative
error of the value is below a certain level (ftol)

GradientCriterion  
The gradient criterion stops the optimization when the gradient at
the current point is less that a given tolerance

AbsoluteValueCriterion  
The absolute criterion stops the optimization when the absolute
error of the value is below a certain level (ftol)

AbsoluteParametersCriterion  
The absolute criterion stops the optimization when the relative
error of the parameters is below a certain level (xtol)

RelativeParametersCriterion  
The relative criterion stops the optimization when the relative
error of the parameters is below a certain level (xtol)

IterationCriterion  
A simple criterion that stops when the iteration limit is reached

MonotonyCriterion  
A simple criterion that stops when the values of the function
starts to rise again

AndComposition  
Compose several criteria with an and rule

OrComposition  
Compose several criteria with an or rule

AICCriterion  
The Akaike information criterion

ModifiedAICCriterion  
The Akaike information criterion with several trials authorized


|  |  |
| --- | --- |
| Functions | [hide private] |

|  |  |
| --- | --- |
| criterion(\*\*kwargs)  Creates a composite criterion based on the formal parameters : | source code |


|  |  |
| --- | --- |
| Variables | [hide private] |

criterion\_\_all\_\_ = `['IterationCriterion', 'MonotonyCriterion',...`


|  |  |
| --- | --- |
| Function Details | [hide private] |

|  |  |
| --- | --- |
| criterion(\*\*kwargs) | source code |

Creates a composite criterion based on the formal parameters :

- iterations\_max indicates the maximum number of iteration
- ftol is the maximum relative change of the value function
- xtol is the maximum relative change of the parameters
- gtol is the maximum gradient

  


|  |  |
| --- | --- |
| Variables Details | [hide private] |

### criterion\_\_all\_\_

Value:
:   ```
    ['IterationCriterion',
     'MonotonyCriterion',
     'RelativeValueCriterion',
     'AbsoluteValueCriterion',
     'RelativeParametersCriterion',
     'AbsoluteParametersCriterion',
     'GradientCriterion',
     'OrComposition',
    ...
    ```

  


| Home | Trees | Indices | Help | PyDSTool |
| --- | --- | --- | --- | --- |

|  |  |
| --- | --- |
| Generated by Epydoc 3.0.1 on Fri May 4 15:24:05 2012 | http://epydoc.sourceforge.net |
